# Seed Morphometry of Native Indonesian Orchids in the Genus *Dendrobium*

**DOI:** 10.1155/2020/3986369

**Published:** 2020-06-20

**Authors:** Sucipto Hariyanto, Intan Ayu Pratiwi, Edy Setiti Wida Utami

**Affiliations:** Department of Biology, Faculty of Science and Technology, Universitas Airlangga, Surabaya 60115, Indonesia

## Abstract

In this study, seeds of 10 species of epiphytic orchids were examined using light and scanning electron microscope. Quantitative and qualitative characters were analyzed. All the presently investigated seeds showed are transparent with visible embryo and remarkable embryo color variations (such as pale yellow, light yellow, shiny yellow to yellow, orange, and white). The species showed two groups in seed shape (fusiform and filiform), prolate and oval-shaped embryo, positioned at the center of the long axis and near apical pole. Embryo in prolate shaped and near apical pole position was only in *D*. *antennatum*. Based on our investigation, there are variations in seed and embryo volume as well as percentage air space in different taxa of orchids. The highest air space percentages were found in *D. leporinum*. According to the ornamentation of testa cells, 3 types of seeds were discovered in this genus. Additionally, the clear variation in the testa ornamentation pattern includes the species of *D. leporinum*, where the testa cells were in the medial regular rectangles, but in the apical and basal pole they are polygonal and irregularly oriented; the testa cells of *D. antennatum* are polygonal and irregularly oriented and those of *D. purpureum* are longitudinally oriented with regular rectangles.

## 1. Introduction

Indonesia has more than 5000 species of orchids spread in Islands of Sumatra, Kalimantan, Jawa, Sulawesi, Maluku, and Papua. Basically, *Dendrobium* is among the largest genera in the orchid family, with 1509 currently described species [1], which mostly grow as epiphytes in tropical and subtropical Asia and Eastern Australia [2–4]. Seed morphology has long been perceived as an important aspect for taxonomic objectives and reflects the evolutionary history of plants [5]. The characteristics used to show morphological diversity in seed included size, shape, and testa surface [6, 7]. These characteristics provide vital information at different taxonomic levels [5, 6]. While some studies [8, 9]support this hypothesis, other investigations show the systematic and taxonomic value of seed micromorphology is limited [10–13]. Besides, seed morphology may affect important biological and ecological aspects such as seed dispersion mechanisms [14].

Variation in seed morphology is an important source of systematic characters for establishing relationships between species within a genus [15–17]. These differences have served as taxonomic and/or phylogenetic markers on seeds of native California orchids and related species [18–21]. Several studies on the morphology of orchid seeds have been carried out including 19 orchids from Turkey [22], genus *Vanilla* [14], *Paphiopedilum* and *Cypripedium* [23], 13 species in tribe Chloraeeae [24], 95 species of 34 genera from the Gulf of Guinea [25], ten *Dendrobium* species using 13 quantitative trait descriptors [26], and other groups in genus *Portulaca* (Portulacaceae) [15]. However, studies on native Indonesian orchid seeds covering morphometry and morphology, especially *Dendrobium*, have not been found. In this study, therefore, a total of 10 species from Indonesia were studied based on their seed morphology and morphometry. These *Dendrobium* species are a collection of DD Orchids Nursery that are used as crosses. The purpose of the study furthermore was to investigate the range of variability regarding seed characteristics in native orchid species to establish their usefulness for future taxonomic works.

## 2. Materials and Methods

### 2.1. Mature Capsules and Seed Collection

The seeds used were 10 species of the *Dendrobium* genus including *D. antennatum*, *D. lineale*, *D. tonson*, *D. odoratum*, *D. discolor*, *D. mirbelianum*, *D. purpureum*, *D. nindii*, *D. affine,* and *D. leporinum* collected from ripe capsules (±3–4.5 months after pollination) during 2015–2018 from DD Orchids Nursery, Batu, and East Java, Indonesia. Fresh seeds were dried for at least 2 weeks and stored in tubes at 5°C in dry conditions.

### 2.2. Observation of Seed Morphology and Micromorphology

Seed samples were observed and photographed under stereomicroscope, light microscope (LM), and scanning electron microscope (SEM). The morphological parameters included seed shape (SS), seed color (SC), seed length (SL), seed width (SW), seed length/seed width (SL/SW), and seed volume (SV). On the basis of embryo, the parameters included embryo shape (ES), embryo color (EC), embryo length (EL), embryo width (EW), embryo length/embryo width (EL/EW), embryo volume (EV), seed volume/embryo volume (SV/EV), and air space (AS) (Tables 1 and 2). Characteristics such as SS, ES, SC, and EC were observed under Tension stereomicroscope, Nikon SMZ-1, Japan. The SC and EC were described in subjective terms while SL, SW, EL, and EW (at the longest and widest axis) were observed using a light microscope (Olympus CH 20, Olympus Japan) and standardized ocular meter. The seed volume (mm^3^ × 10^−3^) was calculated using the formula 2 [(^*L*^/2) (^*W*^/2)^2^ (^*π*^/3) ], where *L* = length, *W* = width, and *π* = 22/7, and the embryo volume (mm^3^ × 10^−3^) was calculated using the formula 4/3 *π* (*L*/2) (*W*/2)^2^, where *L* = length and *W* = width, adapted from Arditti et al. [20]. The values for SL, SW, EL, and EW were recorded from approximately 30 seeds per species. Air space (%) was calculated using the formula ((seed volume−embryo volume)/(seed volume)) × 100%, adapted from Arditti and Ghani [27]. In this study, the quantitative data were analyzed statistically using analysis of variance (ANOVA) and Duncan's test with SPSS 21.0 for Windows.

### 2.3. SEM Study

For SEM preparations, the samples were mounted on SEM stubs and sputter-coated with palladium/gold (SEM coating system SC 7620 Mini Sputter Coater). Detailed seed coat (testa cells) surface was examined with the Generation 5 Phenom Prox SEM, with a filament voltage of 15 kV. The considered parameters were seed coat sculpturing and thickenings.

## 3. Results

### 3.1. Seed Shape and Testa Cells

**Figure 1:**
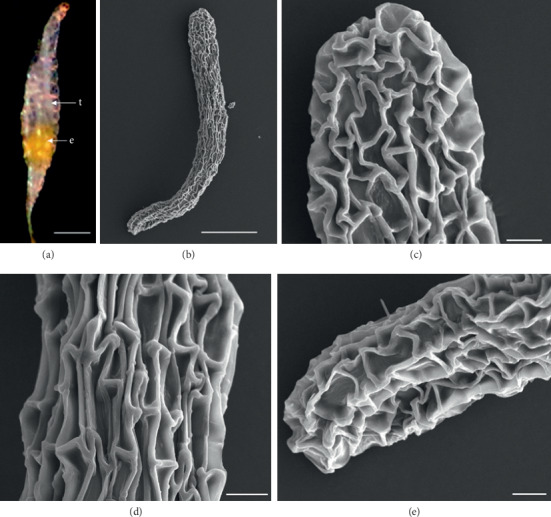
LM and SEM photographs of *D. antennatum.* (a) A seed under LM; (b) a seed under SEM; (c) cells of the chalazal pole; (d) pattern of testa cells of the medial; (e) cells of the micropylar pole. e = embryo, t = testa. Scale bars: (a) 175 *μ*m, (b) 100 *μ*m, and (c–e) 10 *μ*m.

**Figure 2:**
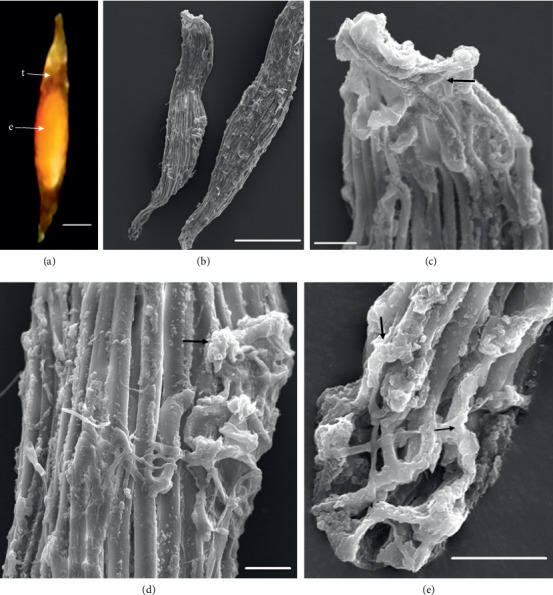
LM and SEM photographs of *D. lineale.* (a) a seed under LM; (b) seeds under SEM; (c) cells of the chalazal pole; (d) pattern of testa cells of the medial; (e) cells of the micropylar pole. e = embryo, t = testa. Scale bars: (a) 81 *μ*m, (b) 100 *μ*m, and (c–e) 10 *μ*m.

**Figure 3:**
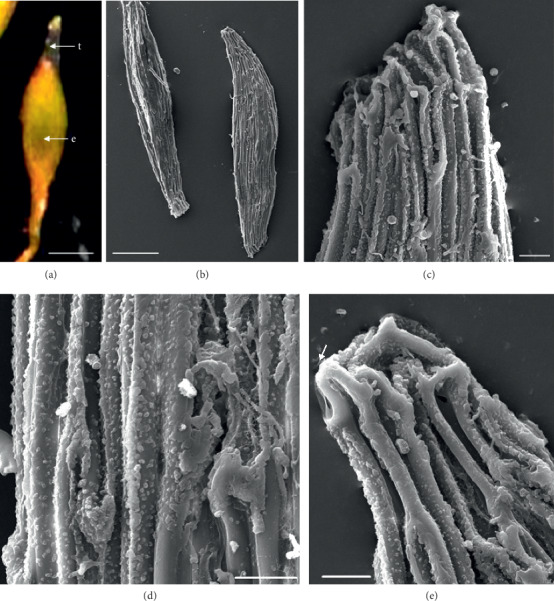
LM and SEM photographs of *D. tonson.* (a) A seed under LM; (b) seeds under SEM; (c) cells of the chalazal pole; (d) pattern of testa cells of the medial; (e) cells of the micropylar pole. e = embryo, t = testa. Scale bars: (a) 85 *μ*m, (b) 100 *μ*m, and (c–e) 10 *μ*m.

**Figure 4:**
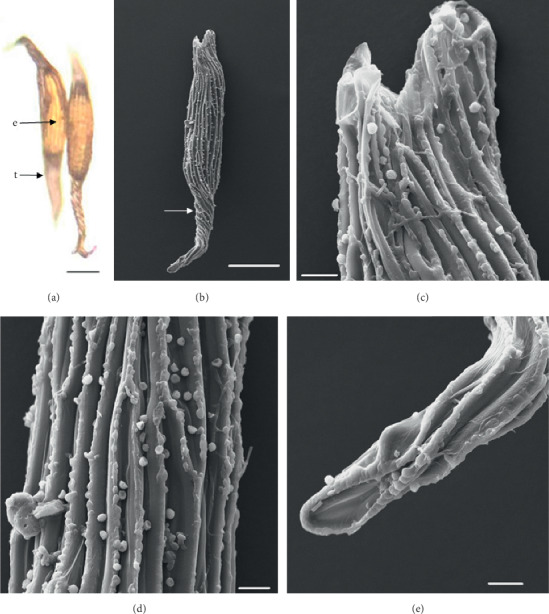
LM and SEM photographs of *D. odoratum.* (a) A seed under LM; (b) a seed under SEM; (c) cells of the chalazal pole; (d) pattern of testa cells of the medial; (e) cells of the micropylar pole. e = embryo, t = testa. Scale bars: (a) 73 *μ*m, (b) 100 *μ*m, and (c–e) 10 *μ*m.

**Figure 5:**
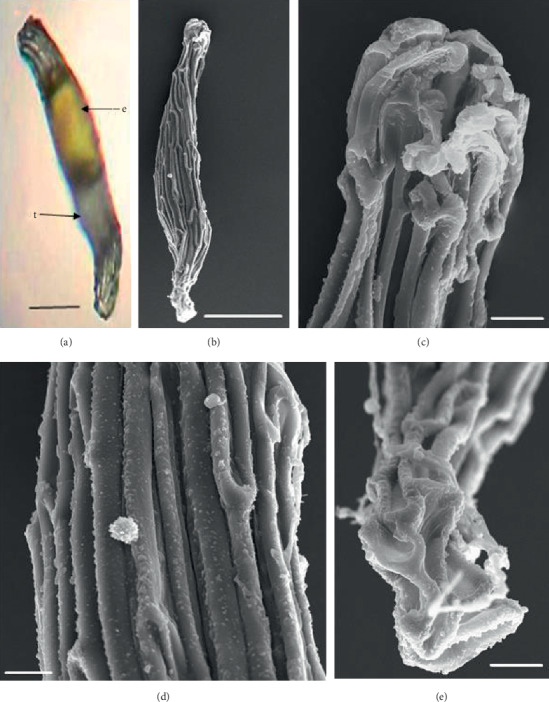
LM and SEM photographs of *D. discolor.* (a) A seed under LM; (b) a seed under SEM; (c) cells of the chalazal pole; (d) pattern of testa cells of the medial; (e) cells of the micropylar pole. e = embryo, t = testa. Scale bars: (a) 78 *μ*m, (b) 100 *μ*m, and (c–e) 10 *μ*m.

**Figure 6:**
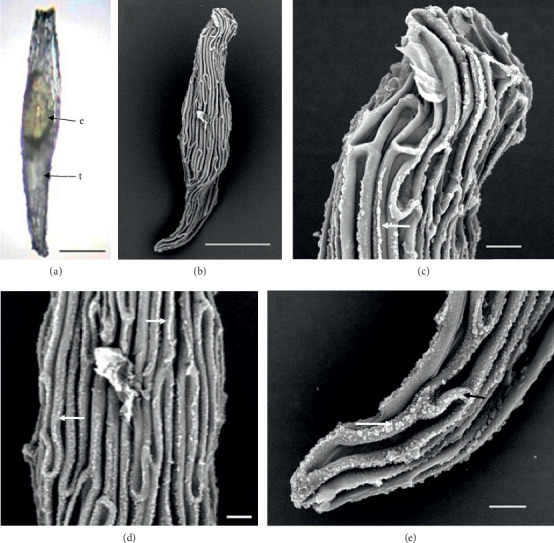
LM and SEM photographs of *D. mirbelianum.* (a) A seed under LM; (b) a seed under SEM; (c) cells of the chalazal pole; (d) pattern of testa cells of the medial; (e) cells of the micropylar pole. e = embryo, t = testa. Scale bars: (a) 82 *μ*m, (b) 100 *μ*m, and (c–e) 10 *μ*m.

**Figure 7:**
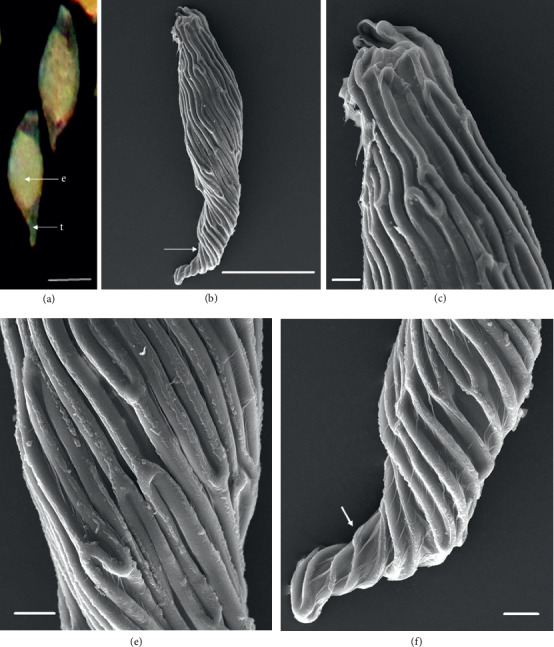
LM and SEM photographs of *D. purpureum.* (a) Seeds under LM; (b) a seed under SEM; (c) cells of the chalazal pole; (d) pattern of testa cells of the medial; (e) cells of the micropylar pole. e = embryo, t = testa. Scale bars: (a) 67 *μ*m, (b) 100 *μ*m, and (c–e) 10 *μ*m.

**Figure 8:**
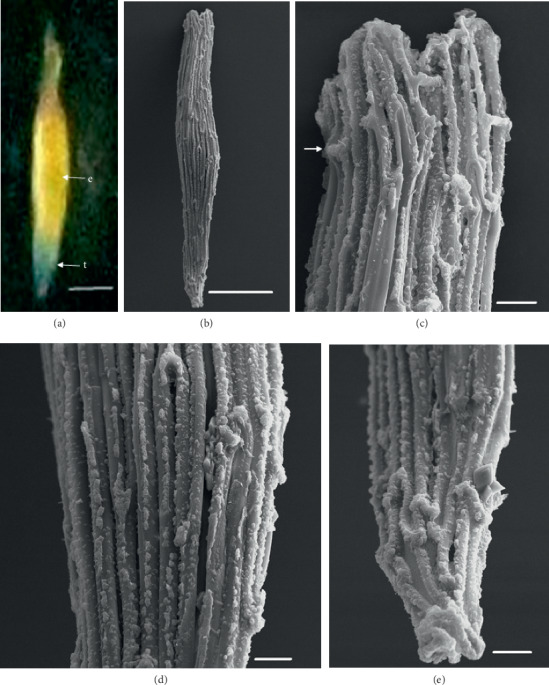
LM and SEM photographs of *D. nindii.* (a) Seed under LM; (b) a seed under SEM; (c) cells of the chalazal pole; (d) pattern of testa cells of the medial; (e) cells of the micropylar pole. e = embryo, t = testa. Scale bars: (a) 75 *μ*m, (b) 100 *μ*m, and (c–e) 10 *μ*m.

**Figure 9:**
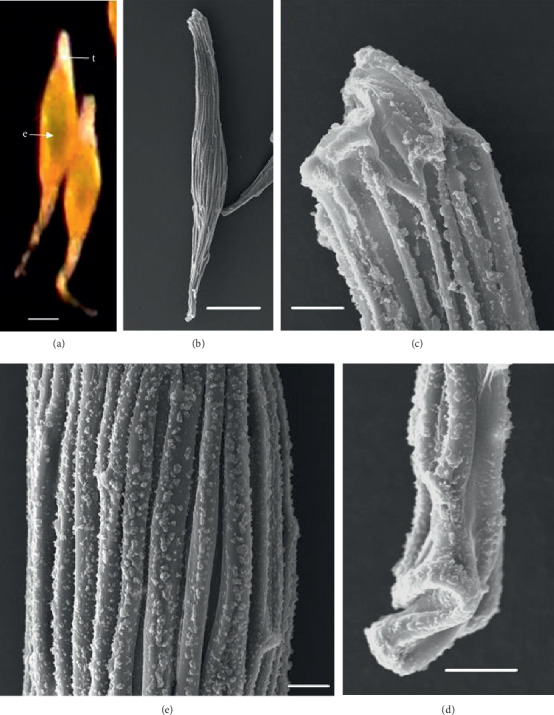
LM and SEM photographs of *D. affine.* (a) Seeds under LM; (b) A seed under SEM; (c) cells of the chalazal pole; (d) pattern of testa cells of the medial; (e) cells of the micropylar pole. e = embryo, t = testa. Scale bars: (a) 85 *μ*m, (b) 100 *μ*m, and (c–e) 10 *μ*m.

**Figure 10:**
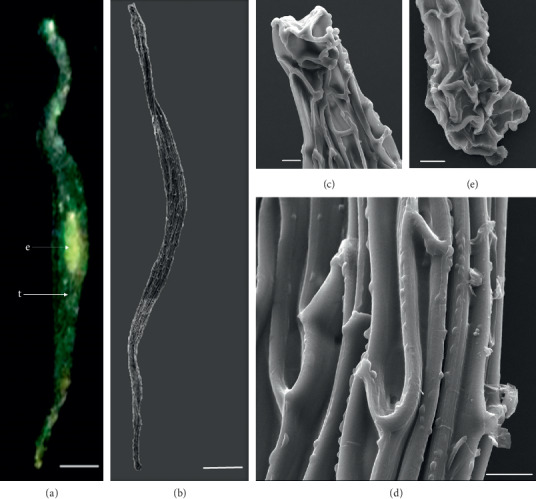
LM and SEM photographs of *D. leporinum.* (a) A seed under LM; (b) a seed under SEM; (c) cells of the chalazal pole; (d) pattern of testa cells of the medial; (e) cells of the micropylar pole. e = embryo, t = testa. Scale bars: (a) 181 *μ*m, (b) 100 *μ*m, and (c–e) 10 *μ*m.

In the 10 species observed, the seeds were generally fusiform in shape except in *D. leporinum,* which is filiform in shape, and the majority had a central embryo position; the seeds of *D. antennatum* are transparent with small embryo located in the near micropylar pole and white color in testa (Figures 1(a) and 1(b)). The testa cells are polygonal and irregularly oriented while the surface is blunt. Testa cell walls were covered with smooth waxes (Figures 1(c), 1(d), and 1(e)). The seeds of *D. lineale* are transparent with a big embryo located at the center (Figures 2(a) and 2(b)). The testa cells are longitudinally oriented with regular rectangles (Figure 2(d)). The testa cell walls were covered with cottony-white substances, and any region of cell walls was covered with thickening white substances (arrow in Figures 2(c), 2(d), and 2(e)). The seeds of *D. tonson* are transparent with a visible embryo located at the center and occupied a major part of the seed space (Figures 3(a) and 3(b). Testa cells are longitudinally oriented with regular rectangles (Figures 3(c), 3(d), and 3(e)). The testa cell walls were covered with bead cottony substances which were thicker at the micropylar pole (arrow in Figure 3(e)). In *D. odoratum*, the seeds are small (Figures 4(a) and 4(b)). Testa cells are longitudinally oriented with regular rectangles, but in the region near the micropylar pole, they are twisted (arrow in Figure 4(b)). Besides, the testa cell walls were with small sphere waxes (Figures 4(c), 4(d), and 4(e)). In *D. discolor*, the seeds are smaller with distinct embryo located at the center of the long axis (Figure 5(a)). Testa cells are longitudinally oriented with regular rectangles and straight on all the seeds (Figure 5(b)). The testa cell walls were covered with cottony-white substances which were more visible at the center (Figures 5(c), 5(d), and 5(e). In the case of *D. mirbelianum*, the seeds have distinct embryo located at the center (Figure 6(a)). Testa cells are longitudinally oriented with regular rectangles and straight (Figure 6(b)). Additionally, the cell walls have smooth wax which was thicker at the center (arrow in Figures 6(c), 6(d), and 6(e)). In *D. purpureum*, the seeds have big and distinct embryos present in the center and occupied a major part of the seed space (Figure 7(a)). Testa cells are spirally oriented with regular rectangles, with twisted rope appearance and more twisted at the micropylar pole (arrow in Figures 7(b) and 7(e)). Moreover, the testa cell walls were covered with smooth cottony-white substances (Figures 7(c), 7(d), and 7(e)). The seeds of *D. nindii* are transparent with the visible embryo, centrally located and occupied a major part of the seed space (Figure 8(a)). Testa cells are longitudinally oriented with regular rectangles (Figure 8(b)). Besides, the cell walls were covered with an adequate thickness of cottony-white substances as cup (arrow in Figure 8(c)) and thick wax in both poles (Figures 8(c) and 8(e)). The seeds of *D. affine* are transparent with the visible embryo located in the center and occupied a major part of the seed space (Figure 9(a). Testa cells are longitudinally oriented with regular rectangles (Figure 9(b)), while the cell walls are covered with dispersed cottony-white substances (Figures 9(c), 9(d), and 9(e)). In the case of *D. leporinum*, the seeds are transparent with the small distinct embryo present at the center (Figures 10(a) and 10(b)). Testa cells in the medial part are longitudinally oriented with regular rectangles (Figure 10(d)), having anticlinal walls with a remarkable deep thickening at the vertex. The testa cells were chalazal and the micropylar pole is polygonal and oriented irregularly (Figures 10(c) and 10(e)), and testa cells were covered with smooth waxes.

### 3.2. Seed Size, Seed Volume, and SL/SW Ratio

Table 1 illustrates the size of seeds from 10 species of the genus *Dendrobium*. Even though the seeds are microscopic, the result of the investigation shows high diversity in their size. The seeds range between 0.353 ± 0.0019 mm and 1.868 ± 0.0128 mm in length and 0.067 ± 0.0018 mm and 0.181 ± 0.0078 mm in width. Seed volume ranges from 0.001 ± 0.000 mm^3^ × 10^−3^ to 7.286 ± 0.1569  mm^3^ × 10^−3^. The higher seed volume is noticed in *D. antennatum* (7.286 ± 0.1569 mm^3^ × 10^−3^), followed by *D. tonson* (1.074 ± 0.0816 mm^3^ × 10^−3^). Other species (*D. lineale, D. odoratum, D. discolor, D. mirbelianum, D. purpureum, D. nindii, D. affine*, *and D. leporinum*) had seeds of lower volume (<1.0). Species with elongated seeds (SL/SW > 6) was observed in *D. tonson*, *D. odoratum*, *D. nindii*, *D. affine*, *and D. leporinum*. Other species (*D. antennatum, D. lineale, D. discolor, D. mirbelianum*, and *D. purpureum*) produced truncated seeds (SL/SW < 6). The maximum SL/SW ratio was noticed in *D. leporinum* (10.315 ± 0.4152 mm) while the minimum was in *D. antennatum* (5.201 ± 0.0740 mm).

Based on the observational data on the size of *Dendrobium* seeds, it is known that *D. leporinum* species are significantly different from other species. *D*. *lineale*, *D*. *discolor*, *D*. *mirbelianum*, *D. purpureum*, and *D. nindii* species are not significantly different from each other (Table 1). This condition is also the same in *D. tonson*, *D*. *odoratum*, and *D*. *affine* species. Another thing that can be known based on statistical testing is that the *D. antennatum* and *D. leporinum* species are significantly different from all the species tested in this study.

### 3.3. Embryo Size, Embryo Volume, and Free Air Space

Embryo color in the observed species varied, i.e., shiny yellow, orange, yellow, pale yellow, light yellow, and white. Shiny yellow embryos are characterized in *D*. *antennatum* (Figure 1(a)), and orange embryos are recorded in *D. lineale* (Figure 2(a)), *D. tonson* (Figure 3(a)), and *D. affine* (Figure 9(a)). Yellow embryos are common in *D. odoratum* (Figure 4(a)). Pale yellow embryos are observed in *D. discolor* (Figure 5(a)) and *D. nindii* (Figure 8(a)). White embryos are found in *D. purpureum* (Figure 7(a)) and *D. leporinum* (Figure 10(a)). Light yellow embryos are characterized in *D. mirbelianum* (Figure 6(a)). In the majority of investigated species, embryos were generally oval-shaped, and only one species has the shape of embryo prolate. Similarly, the results of investigations on the position of the embryo showed that, of the 10 species observed, 9 species had a central embryo position, noticed in *D. lineale* (Figure 2(a))*, D. tonson* (Figure 3(a))*, D. odoratum* (Figure 4(a))*, D. discolor* (Figure 5(a))*, D. mirbelianum* (Figure 6(a)), *D. purpureum* (Figure 7(a)), *D. nindii* (Figure 8(a))*, D. affine* (Figure 9(a)), and *D. leporinum* (Figure 10(a)), but in *D. antennatum*, the position of the embryo was near the micropylar pole (Figure 1(a)).

Observation data on the size of the embryo indicate that the percentage of air space from *leporinum* is the highest. However, the length of the *D*. *leporinum* embryo does not differ significantly from the *D. lineale*, *D. tonson*, and *D. odoratum* (Table 2). Even in the case of the weight of the embryo, *D*. *leporinum* is not significantly different from the *D. antennatum*. This shows that *D*. *leporinum* with almost the same size and weight has a large air space and can enhance subsequent biological processes.

## 4. Discussion

According to Vij et al. [28], Dressler [29], and Molvray and Kores [30], the shape of orchid seeds vary and could be ellipsoid, oblongoid, ovoid, globose, filamentous, spindle, irregular, fusiform, or filiform. The seeds observed in this study were fusiform, except *D. leporinum* which is filiform in shape. According to Molvray and Chase [31], that typical seeds of Orchidoideae were fusiform and ovoid.

With concern about seed coat morphology, reticulations were present on the testa surface of all the *Dendrobium* observed here, although their shapes varied among species. Our examinations that testa cell walls of all seeds were smooth and have epicuticular waxes differed among species. Types of the testa cell walls are specific traits of certain species; nevertheless, these species are phylogenetically distant species included to distinct morphological sections [32].

Seed length/seed width (SL/SW) ratio of the seed was observed highest in *D. leporinum* (10.315 ± 0.4152 mm) and significantly different from other species. Information on the relative degree of truncation of orchid seed is ratio SL/SW [20, 30]. According to Arditti et al. [19], the relative degree of truncation of orchid seeds is associated with augmentation in their length rather than in their width.

From statistical analysis, there are five groups that are significantly different in seed volume. In the species of *D. antennatum*, the highest seed volume is the result of long width to some extent than the length of testa [30, 31].

EL/EW ratio was observed highest in *D. odoratum* (4.208 ± 0.114 mm^3^ × 10^−3^). On the other hand, the EL/EW ratio was observed lowest in *D. antennatum* (1.299 ± 0.024 mm3 × 10^−3^) (Table 2). According to Healey et al. [21], the size of orchid embryos in the same genus tends to be uniform, but in this study, the size of the embryo varies, smallest (0.335 ± 0.017 mm^3^ × 10^−3^) in *D. purpureum* and biggest (0.927 ± 0.036 mm^3^ × 10^−3^) in *D. antennatum* and (0.922 ± 0.125 mm^3^ × 10^−3^) in *D. leporinum*.

According to Arditti et al. [20], Augustine et al. [32] and Swamy et al. [33] emphasize the importance of observing the air space in orchid seeds. The existence of air space in orchid seeds is a reflection of the condition the seeds show the main mode of seed dispersal. In the present investigation, higher percentage of air space of *D. leporinum, D. antennatum*, and D. *odoratum* shown is more than 50%, i.e., (94.245 ± 0.874%), (87.273 ± 0.621%), and (55.269 ± 3.688%). The ratio of SV/EV shown is more than two which is also noticed in *D. leporinum* (17.758 ± 2.630 mm^3^ × ^−3^), *D. antennatum* (7.875 ± 0.374 mm^3^ × 10^−3^), and *D. odoratum* (2.250 ± 0.187 mm^3^ × 10^−3^). In fact, seeds with a greater percentage of air space as in *D. leporinum, D. antennatum*, and *D. odoratum* indicate that it makes the seed light and float, so that it is easily carried away by the wind, so it may get dispersed over wide geographical areas. The other seven species, namely, *D. lineale, D. tonson, D. discolor, D. mirbelianum, D. purpureum*, *D. nindii*, and *D. affine*, have seeds with air spaces below 40% (Table 2). With the result that the seven species may get confined to a few narrow distribution in nature, which will potentially be endemic. If these species become endangered, conservational measures are more difficult. According to Arditti and Ghani [27] and Arditti and Ernst [34], orchid embryos are very small, simple, and only composed of several cells, and most do not have endosperms, generally oval or spherical. The percentage of air inside seed directly involves embryo volume, and consequently, it has a key role in seed dispersal and species dispersion.

## 5. Conclusion

The results of this study show that orchid seeds vary in micromorphology, size, ultrastructure features, and finer detail. Importantly, the characteristics of seeds are used in explaining taxonomic, phylogenetic, and phytogeographic relationships between different orchid taxa. The seeds with the percentage of air space below 49% were found in *D. lineale*, *D. tonson*, *D. discolor*, *D. mirbelianum*, *D. purpureum*, *D. nindii*, and *D. affine*. This implies that the seeds are limited in distribution, hence potentially endemic species. In case the habitat of these 7 species is not properly maintained, their existence is threatened.

## Figures and Tables

**Table 1:** Shape and measurement data of seeds (genus *Dendrobium* Sw).

No	Species	Shape	SL (mm)	SW (mm)	SL/SW (mm)	Volume (mm^3^ x 10^−3^)
1	*D. antennatum*	Fusiform	0.910 ± 0.0071^c^	0.175 ± 0.0019^b^	5.201 ± 0.0740^a^	7.286 ± 0.1569^e^
2	*D. lineale*	Fusiform	0.480 ± 0.0061^a^	0.081 ± 0.0003^a^	5.894 ± 0.0782^a^	0.001 ± 0.0000^a^
3	*D. tonson*	Fusiform	0.564 ± 0.0034^b^	0.085 ± 0.0032^a^	6.624 ± 0.2465^b^	1.074 ± 0.0816^d^
4	*D. odoratum*	Fusiform	0.551 ± 0.0037^b^	0.073 ± 0.0028^a^	7.513 ± 0.2696^b^	0.779 ± 0.0614^c^
5	*D. discolor*	Fusiform	0.405 ± 0.0044^a^	0.078 ± 0.0030^a^	5.220 ± 0.1965^a^	0.642 ± 0.0509^c^
6	*D. mirbelianum*	Fusiform	0.439 ± 0.0086^a^	0.082 ± 0.0045^a^	5.367 ± 0.2971^a^	0.774 ± 0.0864^c^
7	*D. purpureum*	Fusiform	0.353 ± 0.0019^a^	0.067 ± 0.0018^a^	5.307 ± 0.1573^a^	0.410 ± 0.0222^b^
8	*D. nindii*	Fusiform	0.486 ± 0.0019^a^	0.075 ± 0.0015^a^	6.462 ± 0.1356^b^	0.719 ± 0.0269^c^
9	*D. affine*	Fusiform	0.518 ± 0.0046^b^	0.085 ± 0.0015^a^	6.110 ± 0.0805^b^	0.975 ± 0.0411^d^
10	*D. leporinum*	Filiform	1.868 ± 0.0128^d^	0.181 ± 0.0078^b^	10.315 ± 0.4152^c^	0.016 ± 0.0014^a^

Values are expressed as ± standard deviation; SL: seed length; SW: seed width. The data followed by the same letter in a Duncan grouping are not significantly different (*α* = 0.05).

**Table 2:** Shape, color, and measurement data of embryos (genus *Dendrobium* Sw).

No	Species	Shape	Color	EL (mm)	EW (mm)	EL/EW (mm)	Volume (mm^3^ x 10^−3^)	SV/EV (mm^3^ x 10^−3^)	AS (%)
1	*D. antennatum*	Prolate	Shiny yellow	0.144 ± 0.002^b^	0.111 ± 0.002^c^	1.299 ± 0.024^a^	0.927 ± 0.036^e^	7.875 ± 0.374^c^	87.273 ± 0.621^d^
2	*D. lineale*	Oval	Orange	0.215 ± 0.003^c^	0.069 ± 0.001^b^	3.104 ± 0.070^e^	0.542 ± 0.023^b^	1.541 ± 0.065^a^	34.978 ± 2.868^b^
3	*D. tonson*	Oval	Orange	0.226 ± 0.006^c^	0.076 ± 0.002^b^	2.970 ± 0.107^d^	0.685 ± 0.049^c^	1.572 ± 0.132^a^	35.962 ± 5.297^b^
4	*D. odoratum*	Oval	Yellow	0.227 ± 0.003^c^	0.054 ± 0.001^b^	4.208 ± 0.114^f^	0.347 ± 0.013^a^	2.250 ± 0.187^b^	55.269 ± 3.668^c^
5	*D. discolor*	Oval	Pale yellow	0.165 ± 0.011^b^	0.077 ± 0.003^b^	2.130 ± 0.159^b^	0.524 ± 0.057^ab^	1.233 ± 0.117^a^	18.414 ± 5.493^a^
6	*D. mirbelianum*	Oval	Light yellow	0.185 ± 0.004^b^	0.082 ± 0.004^b^	2.264 ± 0.138^b^	0.652 ± 0.070^c^	1.187 ± 0.036^a^	15.679 ± 2.571^a^
7	*D. purpureum*	Oval	White	0.145 ± 0.003^b^	0.067 ± 0.002^b^	2.173 ± 0.088^b^	0.335 ± 0.017^a^	1.223 ± 0.028^a^	18.161 ± 1.800^a^
8	*D. nindii*	Oval	Pale yellow	0.186 ± 0.001^b^	0.070 ± 0.003^b^	2.674 ± 0.121^c^	0.473 ± 0.043^a^	1.534 ± 0.147^a^	34.237 ± 6.206^b^
9	*D. affine*	Oval	Orange	0.084 ± 0.002^a^	0.034 ± 0.002^a^	2.496 ± 0.133^c^	0.750 ± 0.038^d^	1.304 ± 0.091^a^	22.979 ± 5.292^a^
10	*D. leporinum*	Oval	White	0.210 ± 0.009^c^	0.091 ± 0.005^c^	2.304 ± 0.146^bc^	0.922 ± 0.125^e^	17.758 ± 2.630^d^	94.245 ± 0.874^e^

Values are expressed as ± standard deviation; EL: embryo length; EW: embryo width; SV: seed volume; EV: embryo volume; AS: air space; percentage air space was calculated as ((seed volume−embryo volume)/(seed volume)) × 100. *N* = 30. The data followed by the same letter in a Duncan grouping are not significantly different (*α* = 0.05).

## Data Availability

The data used to support the findings of this study are included within the article.
